# Inhibition of P53/miR‐34a improves diabetic endothelial dysfunction via activation of SIRT1

**DOI:** 10.1111/jcmm.14253

**Published:** 2019-02-22

**Authors:** Junduo Wu, Wenzhao Liang, Yueli Tian, Fuzhe Ma, Wenlin Huang, Ye Jia, Ziping Jiang, Hao Wu

**Affiliations:** ^1^ Department of Cardiology The Second Hospital of Jilin University Changchun Jilin China; ^2^ Key Laboratory of Myocardial Ischemia, Ministry of Education Harbin Medical University Harbin Heilongjiang China; ^3^ Department of Neurology China‐Japan Union Hospital of Jilin University Changchun Jilin China; ^4^ Occupational and Environmental Medicine Center Linköping University Linköping Sweden; ^5^ Department of Gastroenteric Medicine The Second Hospital of Jilin University Changchun Jilin China; ^6^ Department of Nephrology The First Hospital of Jilin University Changchun Jilin China; ^7^ School of Science and Technology Georgia Gwinnett College Lawrenceville Georgia; ^8^ Department of Diabetes Complications and Metabolism Diabetes Metabolism Research Institute, Beckman Research Institute of City of Hope Duarte California; ^9^ Department of Hand and Foot Surgery The First Hospital of Jilin University Changchun Jilin China; ^10^ Department of Toxicology and Nutrition, School of Public Health Shandong University Jinan Shandong China

**Keywords:** aorta, diabetes, endothelial dysfunction, miR‐34a, P53

## Abstract

Endothelial dysfunction contributes to diabetic macrovascular complications, resulting in high mortality. Recent findings demonstrate a pathogenic role of P53 in endothelial dysfunction, encouraging the investigation of the effect of P53 inhibition on diabetic endothelial dysfunction. Thus, high glucose (HG)‐treated endothelial cells (ECs) were subjected to pifithrin‐α (PFT‐α)—a specific inhibitor of P53, or *P53*‐small interfering RNA (siRNA), both of which attenuated the HG‐induced endothelial inflammation and oxidative stress. Moreover, inhibition of P53 by PFT‐α or *P53*‐siRNA prohibited P53 acetylation, decreased microRNA‐34a (miR‐34a) level, leading to a dramatic increase in sirtuin 1 (SIRT1) protein level. Interestingly, the miR‐34a inhibitor (miR‐34a‐I) and PFT‐α increased SIRT1 protein level and alleviated the HG‐induced endothelial inflammation and oxidative stress to a similar extent; however, these effects of PFT‐α were completely abrogated by the miR‐34a mimic. In addition, SIRT1 inhibition by EX‐527 or *Sirt1*‐siRNA completely abolished miR‐34a‐I's protection against HG‐induced endothelial inflammation and oxidative stress. Furthermore, in the aortas of streptozotocin‐induced diabetic mice, both PFT‐α and miR‐34a‐I rescued the inflammation, oxidative stress and endothelial dysfunction caused by hyperglycaemia. Hence, the present study has uncovered a P53/miR‐34a/SIRT1 pathway that leads to endothelial dysfunction, suggesting that P53/miR‐34a inhibition could be a viable strategy in the management of diabetic macrovascular diseases.

## 1 INTRODUCTION

Macrovascular complications develop in over a half of the diabetic individuals, leading to high mortality.^1^, ^2^ Endothelial dysfunction is the critical first step of diabetic macrovascular complications.^3^, ^4^ It is therefore essential to improve diabetic endothelial dysfunction, which could prevent or slowdown the development of diabetic macrovascular complications.

Recent findings shed light on a critical role of P53 in endothelial dysfunction.^5^, ^6^ P53 is highly expressed in the endothelium/endothelial cells (ECs) under hyperglycaemia/high glucose (HG) conditions.^5^, ^6^ Acetylation of P53 is critical for its stabilization and function.^8^ We and others have observed hyperacetylation of P53 in the HG‐treated ECs and in the aortas of diabetic mice.^5^, ^9^ This effect enhanced endothelial oxidative stress and inflammation, resulting in endothelial senescence and dysfunction.^5^, ^9^ Notably, deacetylation of P53 by sirtuin 1 (SIRT1) mitigated the HG‐induced endothelial oxidative stress and inflammation,^5^ and improved endothelial dysfunction.^5^, ^9^ On the contrary, forced activation of P53 by nutlin3a increased aortic contractility in healthy mice and generated endothelial oxidative stress and inflammation in both the normal glucose (NG)‐cultured ECs and the aortas of the healthy mice, demonstrating that P53 plays a crucial pathogenic role in endothelial dysfunction.^5^ Collectively, these findings provide a rationale for investigating the effect of P53 inhibition on diabetic endothelial dysfunction.

Despite the identification of P53's pathogenic effect on endothelial dysfunction, little is known for the mechanism by which P53 induces endothelial dysfunction. P53 closely correlates with SIRT1 which functions to protect against diabetic endothelial dysfunction.^5^, ^9^ SIRT1 deacetylates P53, inactivating the transcription of P53‐dependent genes,^10^, ^11^ including *miR‐34a*.^13^, ^14^ In the cytoplasm, miR‐34a directly targets *Sirt1* mRNA without degrading *Sirt1* mRNA, inhibiting SIRT1 protein production.^13^, ^14^ Previously, we found that the protein levels of P53 and acetylated P53 (ac‐P53) were significantly elevated by HG in ECs, accompanied by a drastic decrease in SIRT1 protein level.^5^ The diabetic mice also had higher ac‐P53 level and lower SIRT1 protein level in the aortas compared with the non‐diabetic control mice.^5^ Therefore, we hypothesized that miR‐34a may mediate P53’s pathogenic effect on diabetic endothelial dysfunction by decreasing SIRT1 protein level.

Although *Sirt1* mRNA is a direct target of miR‐34a,^13^, ^14^ it is still needed to investigate whether or not SIRT1 is a major target of miR‐34a in diabetic endothelial dysfunction, given that miR‐34a may target multiple mRNAs.^16^ To this end, inhibition of SIRT1 was achieved by utilizing siRNA‐induced gene silencing and the SIRT1 inhibitor EX‐527, in the presence of the specific inhibitor of miR‐34a (miR‐34a‐I).

In summary, the present study aims to explore: (a) whether or not inhibition of P53/miR‐34a attenuates diabetic endothelial dysfunction; (b) whether or not miR‐34a mediates P53's pathogenic effect; and (c) whether or not SIRT1 is a major target of miR‐34a in diabetic endothelial dysfunction.

## 2 MATERIALS AND METHODS

### 2.1 Animal housing and experiments

C57BL/6 mice were housed in the Animal Center of Jilin University at 22°C, on a 12:12‐hour light‐dark cycle, with free access to rodent feed and tap water. The Institutional Animal Care and Use Committee at Jilin University approved all the experimental procedures, which complied with National Institutes of Health guide for the care and use of Laboratory animals (NIH Publications No. 8023, revised 1978). Eight‐week‐old male mice received intraperitoneal injection of sodium citrate or streptozotocin (50 mg/kg/day, dissolved in 0.1 mol/L sodium citrate, pH 4.5; Sigma‐Aldrich, Shanghai, China) once every day, for five consecutive days.^17^, ^18^ Fasting glucose levels (4‐hour fast) were determined 1 week after the last injection. Mice with fasting glucose levels above 13.89 mmol/L were considered diabetic. Blood glucose was recorded on days 0, 140, 147, 154, 161 and 168, post‐diabetes mellitus (DM) onset.

To study the effect of P53/miR‐34a inhibition on aortic endothelial dysfunction under DM, pifithrin‐α (PFT‐α, 1.1 mg/kg, intraperitoneally injected three times weekly 21, 22; MedChem Express, Shanghai, China) or miR‐34a‐I (2 mg/kg, subcutaneously injected once weekly 23; Thermo Fisher, Shanghai, China) was delivered to the diabetic mice immediately after DM was confirmed, for 24 weeks. In order to investigate the role of SIRT1 in mediating miR‐34a‐I's action, the diabetic mice were treated with EX‐527 (2 mg/kg,^24^ intraperitoneally injected three times weekly; MedChem Express) in the presence of miR‐34a‐I, for 24 weeks. At the end of the procedures, the mice were killed under anaesthesia by intraperitoneal injection of chloral hydrate (0.3 mg/kg),^25^ with their aortas harvested for analysis.

### 2.2 Analysis of aortic dysfunction

Aortic contractility in response to phenylephrine (PE) and relaxation in response to acetylcholine (ACh) were recorded using thoracic aorta, as previously described.^4^, ^5^ Phenylephrine and ACh were administered at doses of 10^−9^, 10^−8^, 10^−7^, 10^−6^, 10^−5^ and 10^−4 ^mol/L.

### 2.3 Analysis of aortic morphology

The freshly harvested thoracic aortas were immediately fixed into 10% buffered formalin solution and were embedded in paraffin, followed by sectioning into 5‐µm‐thick sections onto glass slides. Haematoxylin and eosin (H&E) staining was performed to evaluate morphological change. The thickness of tunica media was measured. Selection of areas to photograph and scoring were done by people blind to the identity of the samples.

### 2.4 Immunohistochemical staining

Immunohistochemical staining was performed as previously described,^26^ using antibodies against ac‐P53 (1:100; Abcam, Shanghai, China), SIRT1 (1:100; Abcam), vascular cell adhesion molecule‐1 (VCAM‐1, 1:100; Santa Cruz Biotechnology, Dallas, TX) and 4‐hydroxynonenal (4‐HNE, 1:100; Alpha Diagnostic Int., San Antonio, TX). Immunohistochemical positive area was quantified within the full‐thickness of the artery wall. Selection of areas to photograph and scoring were done by people blind to the identity of the samples.

### 2.5 Cell culture and experiments

Endothelial cells were isolated from the aortas of 8‐week‐old C57BL/6 male mice, as previously described.^4^, ^5^, ^27^ To investigate the impact of HG on P53/miR‐34a/SIRT1 expression, NG (1 g/L)‐cultured ECs were subjected to mannitol or HG (4.5 g/L), for 48 hours. In order to study the effect of P53 inhibition on the expression of P53/miR‐34a/SIRT, inflammatory genes and oxidative stress, HG‐stimulated ECs were co‐treated with *P53*‐siRNA (20 nmol/L 5, 28; GenePharma, Suzhou, Jiangsu, China), or PFT‐α (20 μmol/L,^22^ MedChem Express), for 48 hours. To investigate the effect of miR‐34a inhibition on inflammation and oxidative stress, HG‐treated ECs were co‐treated with miR‐34a‐I, in parallel with PFT‐α, for 48 hours. The role of miR‐34a in mediating P53’s action was further tested using miR‐34a mimic (miR‐34a‐M) in the presence of PFT‐α, under the HG condition. The HG‐stimulated ECs were treated with miR‐34a‐I, in the presence of either *Sirt1*‐siRNA (20 nmol/L 29; GenePharma) or EX‐527 (2 μmol/L 30; MedChem Express), with the aim of investigating the role of SIRT1 in mediating miR‐34a‐I's protective effect.

### 2.6 Quantitative Real‐time PCR

Quantitative Real‐time PCR was performed using aortic tissue and cell lysate as previously described.^23^, ^26^ The primers for glyceraldehyde‐3‐phosphate dehydrogenase (*Gapdh*), *Icam‐1*, *pre‐miR‐34a*, *miR‐34*a, monocyte chemoattractant protein 1 (*Mcp‐1*), *P53*, selectin E (*Sele*), *Sirt1*, *U6* and *Vcam‐1* were obtained from Life Technologies (Shanghai, China).

### 2.7 Western blot

Western blot analysis was performed using cell lysates, as described in our previous studies,^5^, ^26^, ^31^ with antibodies against ac‐P53 (1:500; Abcam), GAPDH (1:3000; Santa Cruz Biotechnology), P53 (1:1000; Cell Signaling Technology, Shanghai, China) and SIRT1 (1:1000; Abcam).

### 2.8 Analysis of reactive oxygen species and lipid peroxides

Reactive oxygen species (ROS) and malondialdehyde (MDA) levels were measured in cell lysates, using assay kits from Nanjing Jiancheng Bioengineering Institute (Nanjing, Jiangsu, China), following the manufacturer's instructions.

### 2.9 Analysis of SIRT1 activity

Sirtuin 1 activity was analysed in cell lysates using a fluorometric assay kit from BioVision (Milpitas, CA), following the manufacturer's instructions.

### 2.10 Statistical analysis

Cell experiments were performed in triplicate. Eight mice per group were studied. Western blot images were analysed by Image Studio Lite (LI‐COR Biosciences, Lincoln, NE). Immunohistochemical positive area was quantified using Image Pro Plus 6.0 software (Media Cybernetics, Rockville, MD). One‐way ANOVA was performed for the comparisons among the groups. The measurements for each group were summarized as means ± SD. A test is significant if *P < *0.05.

## 3 RESULTS

### 3.1 HG increased P53 and miR‐34a levels and inhibited *Sirt1* expression in ECs

**Figure 1:**
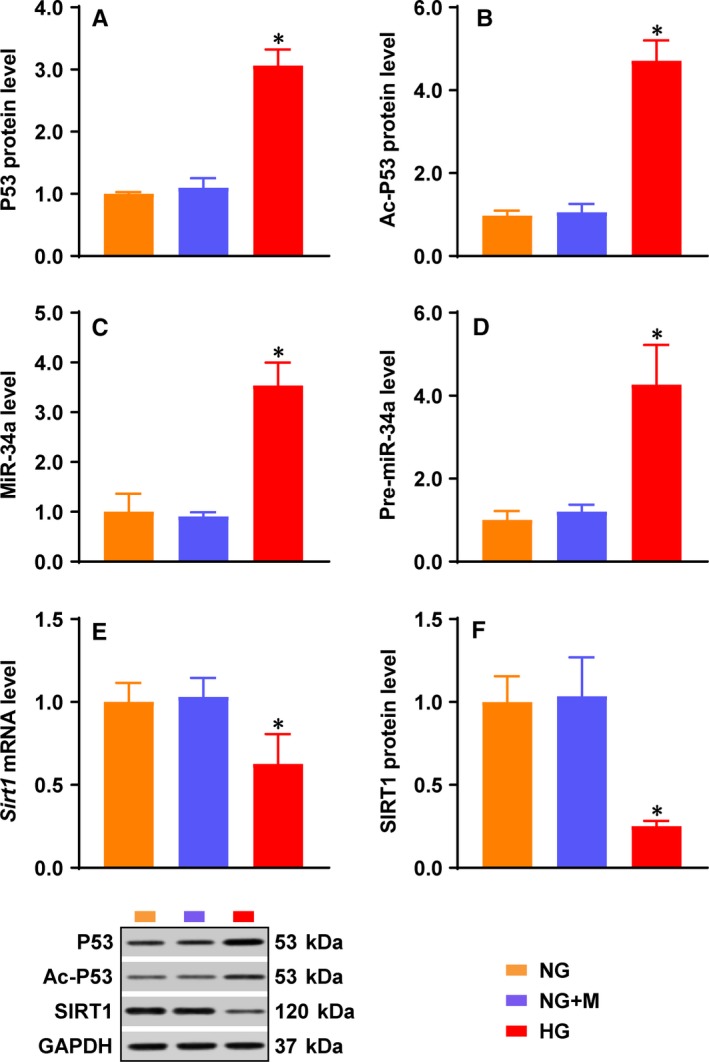
HG increased P53 and miR‐34a levels and inhibited *Sirt1* expression in ECs. NG‐cultured ECs were treated with mannitol or HG. Protein levels of (A) P53 and (B) ac‐P53 were determined by Western blot. C, Pre‐miR‐34a and (D) miR‐34a levels were measured by qPCR *Sirt1*(E) mRNA and (F) protein levels were determined by qPCR and Western blot respectively. GAPDH was used as an endogenous control for P53, ac‐P53 and *Sirt1* expression. U6 was used as an endogenous control for pre‐miR‐34a and miR‐34a. The data were normalized to NG and are presented as means ± SD (n = 3). *P < 0.05 vs NG. Bars: orange, NG; blue, NG + M; red, HG. Abbreviations: ac‐P53, acetylated P53; EC, endothelial cell; GAPDH, glyceraldehyde‐3‐phosphate dehydrogenase; HG, high glucose; M, mannitol; SIRT1, sirtuin 1

To determine whether or not P53/miR‐34a/SIRT1 pathway is altered under HG condition, ECs were treated with NG, NG + mannitol or HG for 48 hours. The protein levels of P53 and ac‐P53 were significantly increased under the HG condition (Figure 1A,B). This led to an enhanced expression of the *miR‐34a* gene, as shown by the increased levels of pre‐miR‐34a and miR‐34a (Figure 1C,D). HG inhibited *Sirt1*expression at both mRNA and protein levels (Figure 1E,F) in ECs.

### 3.2 Inhibition of P53 attenuated the HG‐induced inflammation and oxidative stress in ECs

**Figure 2:**
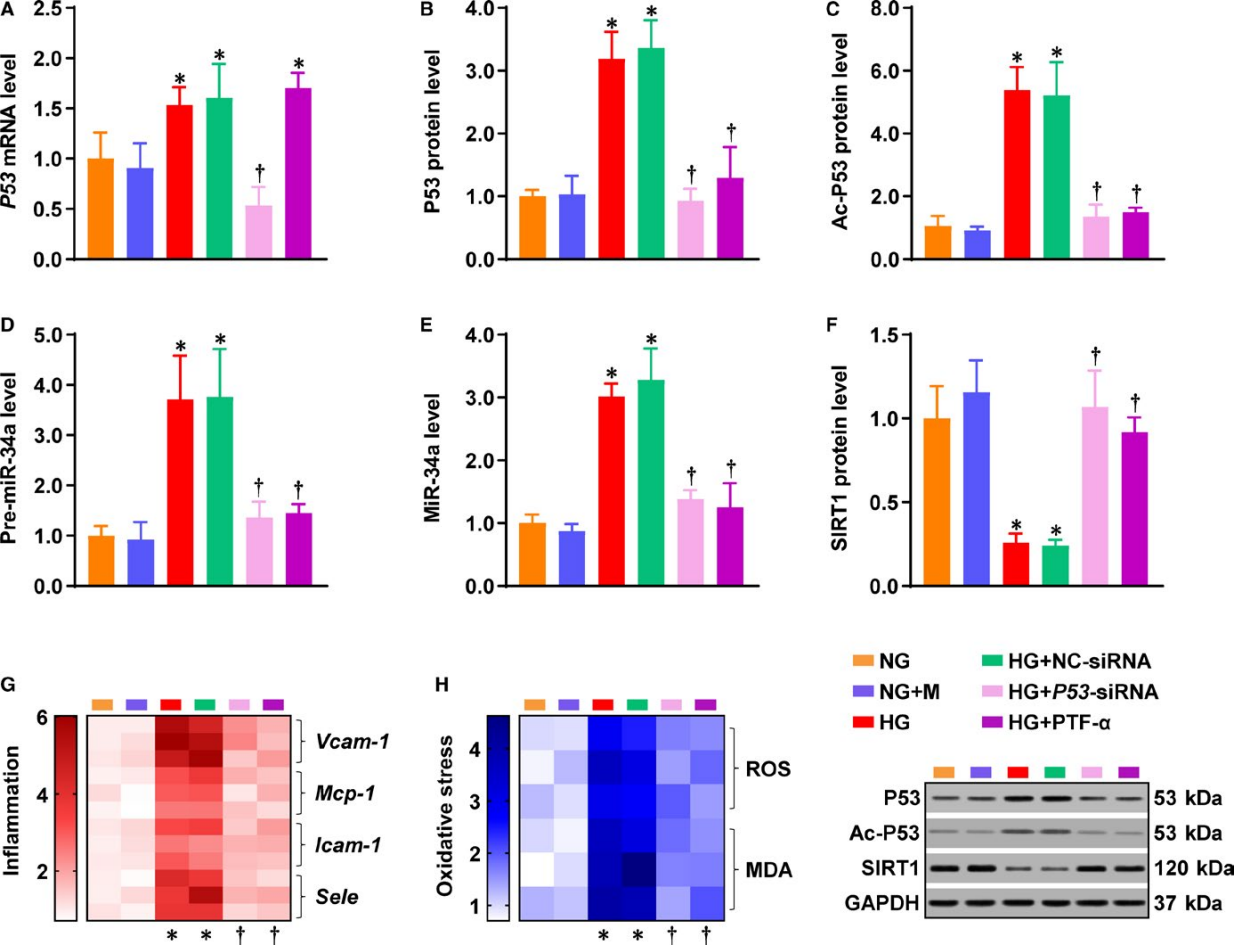
Inhibition of P53 attenuated the HG‐induced inflammation and oxidative stress in ECs. NG‐cultured ECs were treated with HG, in the presence of PFT‐α, *P53*‐siRNA or its negative control siRNA. The levels of (A) P53 mRNA, (B) P53 protein, (C) ac‐P53, (D) pre‐miR‐34a, (E) miR‐34a and (F) SIRT1 protein were determined in all the groups. G, Endothelial inflammation was assessed by determining mRNA levels of *Vcam‐1*, *Mcp‐1*, *Icam‐1*and *Sele*, as summarized in the heat map. H, Heat map for ROS and MDA. GAPDH was used as an endogenous control for *P53*, ac‐P53, SIRT1, *Vcam‐1*, *Mcp‐1*, *Icam‐1* and *Sele* expression. U6 was used as an endogenous control for pre‐miR‐34a and miR‐34a. The data were normalized to NG and are presented as means ± SD (n = 3). *P < 0.05 vs NG. ^†^ *P < *0.05 vs HG. Bars: orange, NG; blue, NG + M; red, HG; green, HG + NC‐siRNA; pink, HG + P53‐siRNA; purple, HG + PFT‐α. Abbreviations: *Icam‐1*, intercellular adhesion molecule‐1; *Mcp‐1*, monocyte chemoattractant protein 1; MDA, malondialdehyde; NC, negative control; PFT‐α, pifithrin‐α; ROS, reactive oxygen species; *Sele*, selectin E; siRNA, small interfering RNA; *Vcam‐1*, vascular cell adhesion molecule‐1. Other abbreviations are the same as in Figure 1

The following study investigated the effect of P53 inhibition on HG‐induced inflammation and oxidative stress in ECs. Inhibition of P53 was achieved by using either *P53*‐siRNA or PFT‐α. *P53*‐siRNA, but not PFT‐α, decreased *P53* mRNA level that was elevated by HG (Figure 2A); however, the protein levels of P53 and ac‐P53 were both significantly decreased by *P53*‐siRNA and PFT‐α (Figure 2B,C). Both *P53*‐siRNA and PFT‐α inhibited *miR‐34a* expression (Figure 2D,E) and rescued SIRT1 protein level that was decreased by HG (Figure 2F). HG treatment induced the mRNA levels of *Vcam‐1*, *Mcp‐1*, *Icam‐1* and *Sele* (Figure 2G), as well as the levels of ROS and MDA (Figure 2H). All these effects were remarkably reduced by *P53*‐siRNA or PFT‐α (Figure 2G,H). These results demonstrate that inhibition of P53 alters miR‐34a and SIRT1 expression, mitigating HG‐induced inflammation and oxidative stress in ECs.

### 3.3 P53's effect on endothelial inflammation and oxidative stress was completely mediated by miR‐34a under the HG condition

**Figure 3:**
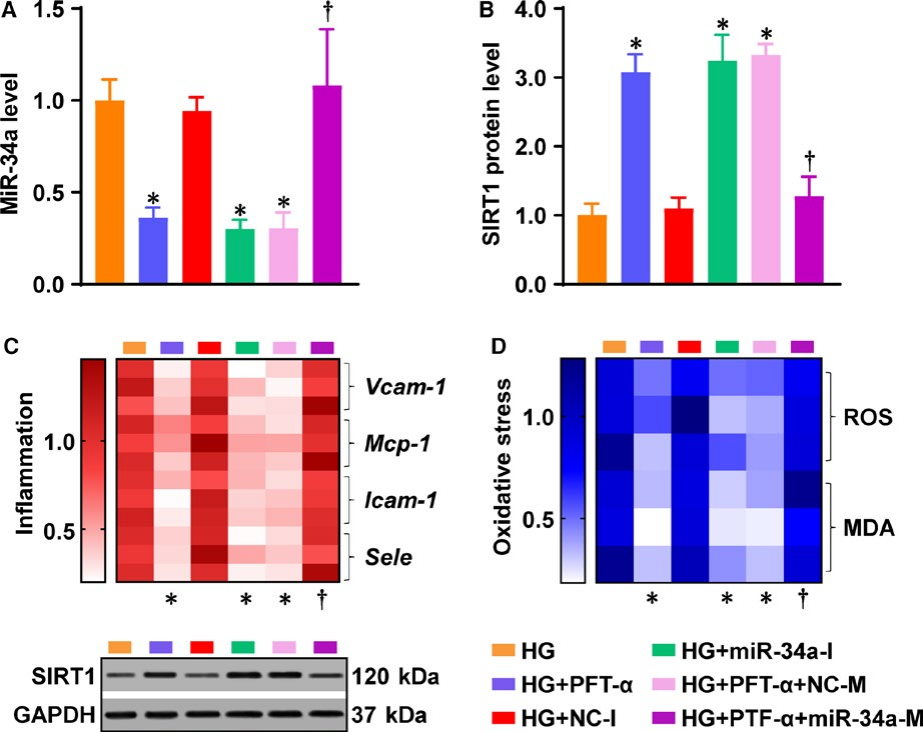
P53's effect on endothelial inflammation and oxidative stress was completely mediated by miR‐34a under the HG condition. HG‐stimulated ECs were treated with NC‐I, miR‐34a‐I, PFT‐α, or PFT‐α in the presence or either NC‐M or miR‐34a‐M. (**A**) MiR‐34a and (B) SIRT1 protein levels were determined. C, Heatmap for mRNA expression of inflammatory genes *Vcam‐1*, *Mcp‐1*, *Icam‐1*and *Sele*. D, Heatmap for oxidative stress indicators ROS and MDA. GAPDH was used as an endogenous control for SIRT1, *Vcam‐1*, *Mcp‐1*, *Icam‐1* and *Sele* expression. U6 was used as an endogenous control for miR‐34a. The data were normalized to HG and are presented as means ± SD (n = 3). *P < 0.05 vs HG. ^†^ *P < *0.05 vs HG + PFT‐α + NC‐M. Bars: orange, HG; blue, HG + PFT‐α; red, HG + NC‐I; green, HG + miR‐34a‐I; pink, HG + PFT‐α + NC‐M; purple, HG + PFT‐α + miR‐34a‐M. Abbreviations: miR‐34a‐I, miR‐34a inhibitor; miR‐34a‐M, miR‐34a mimic; NC‐I, negative control for miR‐34a‐I; NC‐M, negative control for miR‐34a‐M. Other abbreviations are the same as in Figures 1 and 2

To investigate whether to what extent miR‐34a mediates P53's effect on endothelial inflammation and oxidative stress under HG, the HG‐treated ECs were co‐treated with PFT‐α, in the presence or absence of miR‐34a‐M. In addition, miR‐34a‐I was tested for its effect on HG‐induced endothelial inflammation and oxidative stress. Strikingly, miR‐34a‐M completely reversed PFT‐α's effects on the expression of miR‐34a/SIRT1 (Figure 3A,B), the mRNA levels of *Vcam‐1*, *Mcp‐1*, *Icam‐1* and *Sele* (Figure 3C), as well as the levels of ROS and MDA (Figure 3D). MiR‐34a‐I produced similar effects on these parameters to the effects of PFT‐α (Figure 3A‐D). Thus, miR‐34a completely mediated P53's action on HG‐induced endothelial inflammation and oxidative stress.

### 3.4 SIRT1 is a major target of miR‐34a in HG‐induced endothelial inflammation and oxidative stress

**Figure 4:**
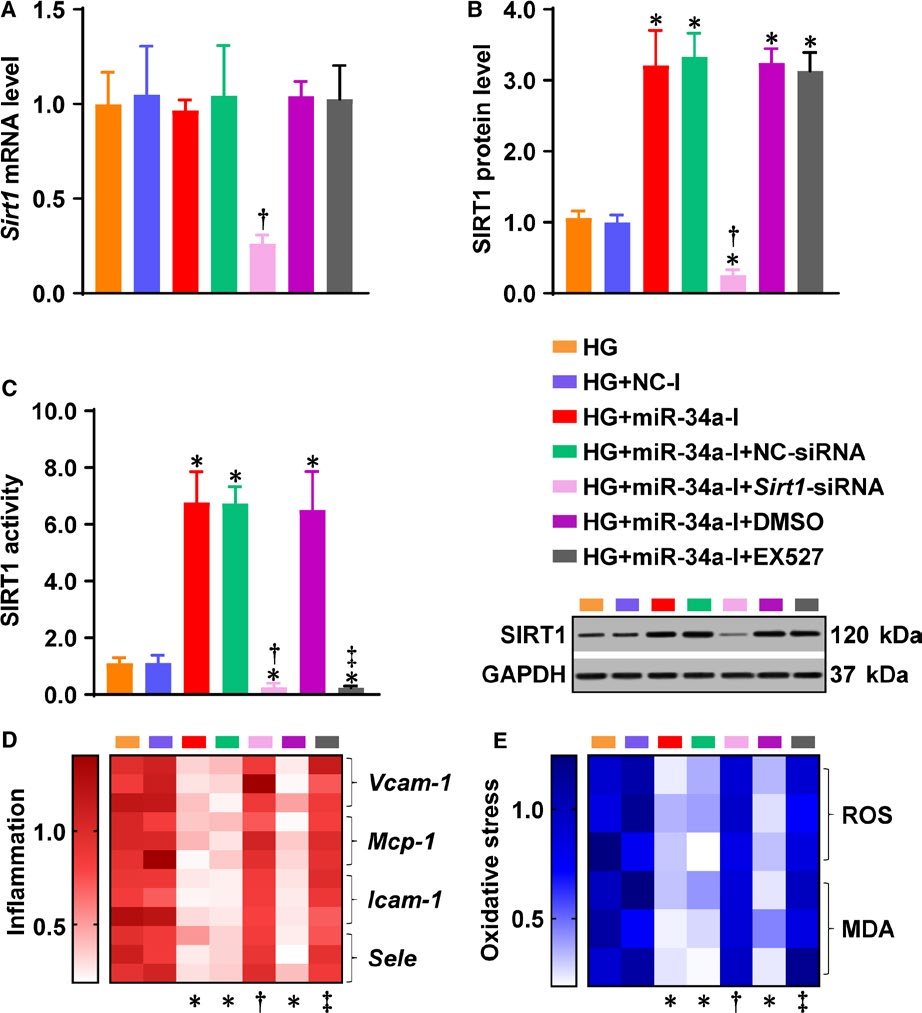
SIRT1 is a major target of miR‐34a in HG‐induced endothelial inflammation and oxidative stress. The effect of SIRT1 inhibition was investigated in the presence of miR‐34a‐I in HG‐treated ECs. A, *Sirt1* mRNA, (B) protein and (C) activity were determined. D, Heatmap for mRNA expression of inflammatory genes *Vcam‐1*, *Mcp‐1*, *Icam‐1*and *Sele*. E, Heatmap for oxidative stress indicators ROS and MDA. GAPDH was used as an endogenous control for *Sirt1*, *Vcam‐1*, *Mcp‐1*, *Icam‐1* and *Sele* expression. The data were normalized to HG and are presented as means ± SD (n = 3). *P < 0.05 vs HG; ^†^ *P < *0.05 vs HG + miR‐34a‐I + NC‐siRNA; ^‡^ *P < *0.05 vs HG + miR‐34a‐I + DMSO. Bars: orange, HG; blue, HG + NC‐I; red, HG + miR‐34a‐I; green, HG + miR‐34a‐I + NC‐siRNA; pink, HG + miR‐34a‐I + *Sirt1*‐siRNA; purple, HG + miR‐34a‐I + DMSO; grey, HG + miR‐34a‐I + EX‐527. Abbreviations are the same as in Figures 1, 2, 3

In order to investigate whether or not SIRT1 is required for miR‐34a's role in mediating HG‐induced endothelial inflammation and oxidative stress, the ECs were treated with HG and miR‐34a‐I, in the presence of either *Sirt1*‐siRNA or EX‐527. *Sirt1*‐siRNA, but not EX‐527, significantly reduced *Sirt1* mRNA and protein levels (Figure 4A,B). However, the miR‐34a‐I‐enhanced SIRT1 activity was remarkably inhibited by both *Sirt1*‐siRNA and EX‐527 (Figure 4C), abolishing the inhibition of inflammation and oxidative stress produced by miR‐34a‐I (Figure 4D,E). These results indicate that SIRT1 is a major target of miR‐34a in HG‐induced endothelial inflammation and oxidative stress.

### 3.5 Inhibition of P53 or miR‐34a attenuated the hyperglycaemia‐induced aortic inflammation and oxidative stress

**Figure 5:**
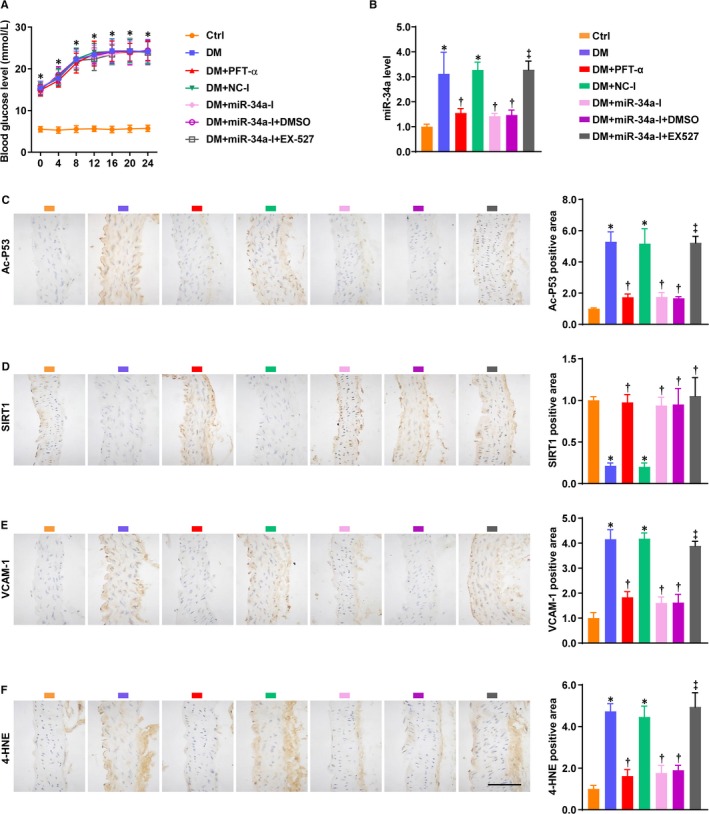
Inhibition of P53/miR‐34a attenuated the hyperglycaemia‐induced aortic inflammation and oxidative stress. C57BL/6 male mice were induced to DM by streptozotocin. The effects of P53/miR‐34a inhibition on the diabetic mice were investigated by treatments with PFT‐α and miR‐34a‐I. The miR‐34a‐I‐treated diabetic mice were co‐treated with EX‐527 to study the role of SIRT1 in mediating miR‐34a's effect. A, Blood glucose levels were recorded 0, 4, 8, 12, 16, 20 and 24 wk post DM onset. B, The level of miR‐34a was determined in the aortas of the mice. U6 was used as an endogenous control for miR‐34a. IHC staining (bar = 100 µmol/L) was performed to detect the protein expression of (C) ac‐P53, (D) SIRT1, (E) VCAM‐1 and (F) 4‐HNE. For (B‐F), the data were normalized to Ctrl and are presented as means ± SD (n = 8). *P < 0.05 vs Ctrl; ^†^ *P < *0.05 vs DM; ^‡^ *P < *0.05 vs DM + miR‐34a‐I + DMSO. Plots and bars: orange, Ctrl; blue, DM; red, DM + PFT‐α; green, DM + NC‐I; pink, DM + miR‐34a‐I; purple, DM + miR‐34a‐I + DMSO; grey, DM + miR‐34a‐I + EX‐527. Abbreviations: 4‐HNE, 4‐hydroxynonenal; Ctrl, control; DM, diabetes mellitus. Other abbreviations are the same as in Figures 1, 2, 3

The following study explored the effect of P53 or miR‐34a inhibition on hyperglycaemia‐induced aortic inflammation and oxidative stress in mice. EX‐527 was administered to the miR‐34a‐I‐treated diabetic mice, with the aim of verifying whether SIRT1 mediates miR‐34a‐I's effect in vivo. Blood glucose level was not affected by PFT‐α, miR‐34a‐I and EX‐527 in the diabetic mice (Figure 5A). In accordance with the effects in ECs, PFT‐α and miR‐34a‐I similarly decreased aortic miR‐34a and ac‐P53 levels, which were significantly elevated by hyperglycaemia (Figure 5B,C). EX‐527 completely abrogated miR‐34a‐I's inhibitory effects on aortic expression of miR‐34a and ac‐P53 (Figure 5B,C). In contrast to miR‐34a and ac‐P53, SIRT1 protein was less expressed in the diabetic aortas compared with the non‐diabetic controls (Figure 5D). This effect was rescued by PFT‐α and miR‐34a‐I (Figure 5D). EX‐527 did not decrease the aortic SIRT1 protein level elevated by miR‐34a‐I (Figure 5D). Further, PFT‐α and miR‐34a‐I reduced the hyperglycaemia‐induced aortic VCAM‐1 and 4‐HNE levels (Figure 5E,F). These effects of miR‐34a‐I were completely reversed by EX‐527. These findings demonstrate the beneficial effects of P53 or miR‐34a inhibition on hyperglycaemia‐induced aortic inflammation and oxidative stress. In addition, SIRT1 was found to predominantly mediate miR‐34a‐I's effects in vivo.

### 3.6 Inhibition of P53/miR‐34a improved the hyperglycaemia‐induced aortic endothelial dysfunction via activation of SIRT1

**Figure 6:**
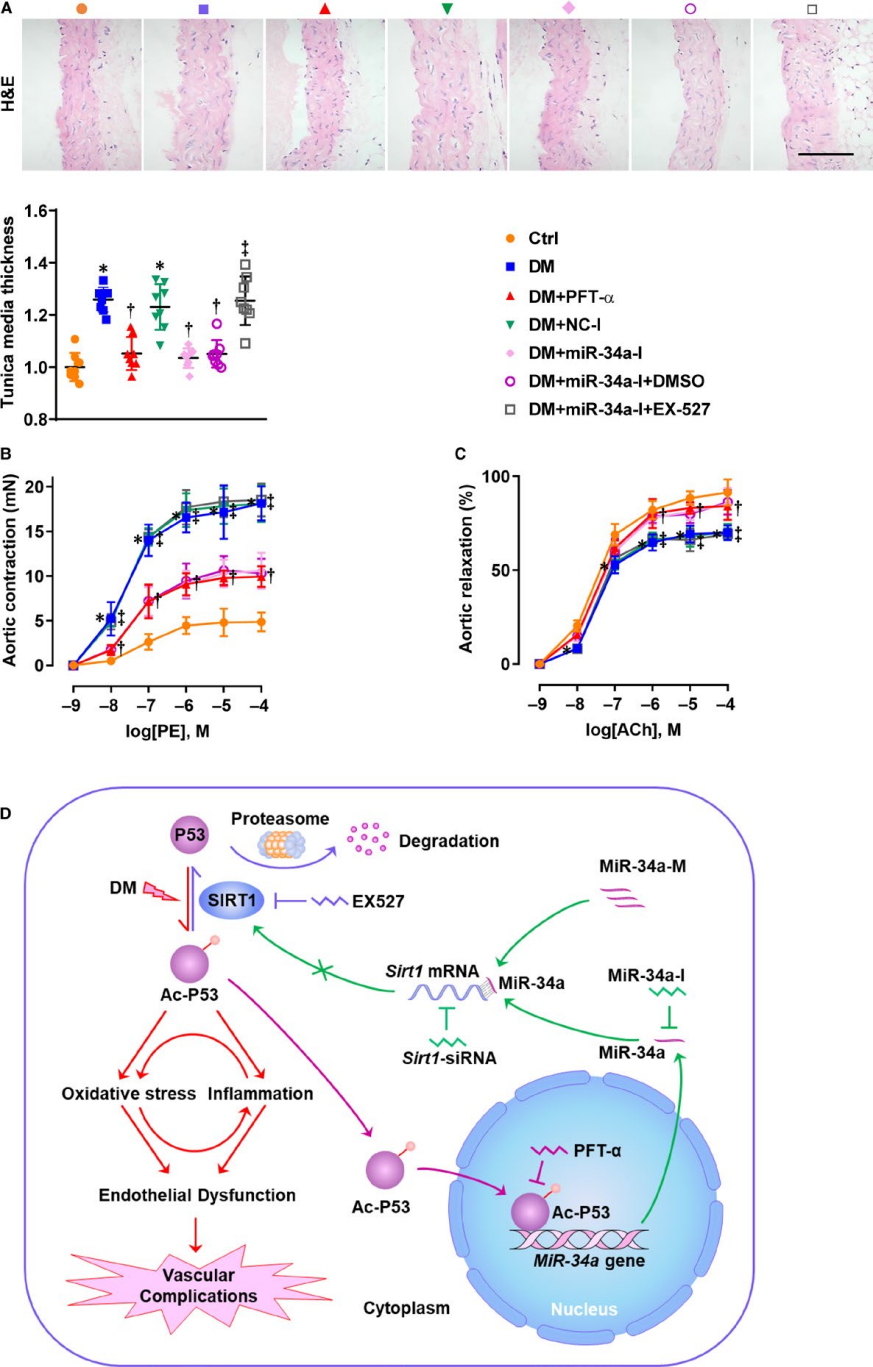
Inhibition of P53 or miR‐34a improved the hyperglycaemia‐induced aortic endothelial dysfunction via activation of SIRT1. A, H&E staining was performed to evaluate aortic morphology, with tunica media thickness measured (bar = 100 µmol/L). B, Aortic contraction in response to PE and (C) relaxation in response to ACh were determined to assess endothelial dysfunction. The doses of PE and ACh were 10^−9^, 10^−8^, 10^−7^, 10^−6^, 10^−5^ and 10^−4^ mol/L. For tunica media thickness, the data were normalized to Ctrl and are presented as means ± SD (n = 8). *P < 0.05 vs Ctrl; ^†^ *P < *0.05 vs DM; ^‡^ *P < *0.05 vs DM + miR‐34a‐I + DMSO. Plots: orange, Ctrl; blue, DM; red, DM + PFT‐α; green, DM + NC‐I; pink, DM + miR‐34a‐I; purple, DM + miR‐34a‐I + DMSO; grey, DM + miR‐34a‐I + EX‐527. D, Schematic diagram: P53 is hyperacetylated under diabetic condition. This elevates oxidative stress and inflammation, leading to endothelial dysfunction which is the critical first step for vascular complications (red pathway). Acetylation is essential for P53's stabilization and function. In the nucleus, ac‐P53 activates the transcription of the *miR‐34a* gene, the effect of which can be prohibited by PFT‐α (purple pathway). Ac‐P53‐directed miR‐34a production inhibits SIRT1 protein translation through binding the 3′ untranslated region of the *Sirt1* mRNA (green pathway). This effect can be modulated by miR‐34a‐I, miR‐34a‐M and *Sirt1*‐siRNA. SIRT1 deacetylates P53, facilitating degradation of P53 (blue pathway). This effect can be reversed by EX527. Therefore, a P53/miR‐34a/SIRT1 circuit may contribute to diabetic endothelial dysfunction. Abbreviations: ACh, acetylcholine; H&E, haematoxylin and eosin; PE, phenylephrine. Other abbreviations are the same as in Figures 1, 2, 3 and 5

Morphological study reviewed no prominent change in the diabetic aortas compared with the non‐diabetic aortas (Figure 6A). However, the diabetic aortas exhibited derangement of endothelial and smooth muscle cells (Figure 6A). Moreover, a mild increase in the thickness of tunica media was observed in the diabetic mice and this was significantly decreased by PFT‐α and miR‐34a‐I (Figure 6A).

To study the effect of P53 or miR‐34a inhibition on endothelial dysfunction, aortic contraction and relaxation were determined in the presence of PE or ACh respectively (Figure 6B,C). The diabetic mice exhibited aortic endothelial dysfunction, as revealed by the significantly enhanced contraction and impaired relaxation (Figure 6B,C). The impaired aortic endothelial dysfunction was remarkably improved by PFT‐α and miR‐34a‐I (Figure 6B,C). Further, EX‐527 completely blocked miR‐34a‐I's effects on aortic dysfunction (Figure 6B,C).

## 4 DISCUSSION

The present study has found a P53/miR‐34a/SIRT1 pathway that contributes to diabetic endothelial dysfunction (Figure 6D). This conclusion is based on the following observations: (a) HG/hyperglycaemia significantly altered endothelial/aortic expression of P53/miR‐34a/SIRT1; (b) Inhibition of P53 reversed the effect of HG on miR‐34a/SIRT1 expression; (c) Inhibition of P53 and miR‐34a mitigated the HG/hyperglycaemia‐induced endothelial/aortic inflammation and oxidative stress and improved the hyperglycaemia‐induced aortic endothelial dysfunction; (d) By using miR‐34a‐M in the presence of PFT‐α, miR‐34a was demonstrated to completely mediate P53's pathogenic actions; (e) By inhibiting SIRT1 in the presence of miR‐34a‐I, miR‐34a was found to act through inhibition of SIRT1 to induce diabetic endothelial dysfunction (Figure 6D).

P53 was primarily known as a guardian of genome in coordinating cellular responses to genotoxic stress.^32^, ^33^ Not until recent years was P53 identified to play a pathogenic role in DM and its complications. P53 is highly expressed under diabetic condition, contributing to the pathogenesis of DM,^34^, ^35^ diabetic cardiomyopathy,^36^ nephropathy^22^, ^37^ and peripheral artery disease.^38^ Here we report the pathogenic effect of P53 on diabetic aortic endothelial dysfunction, with miR‐34a to be the mediator. Like P53, miR‐34a was reported to promote multiple complications of DM, including nephropathy, endothelial dysfunction and neuropathy.^39^, ^40^ As P53/miR‐34a activation leads to DM and its complications, inhibition of P53/miR‐34a may produce beneficial effects on multiple organs/tissues in diabetic individuals. This ‘one stone for multiple birds’ strategy has a unique advantage in future clinical management of DM and its complications.

The present study has found an unhindered effect passing from P53 to the endothelium through miR‐34a and SIRT1, by supplementing miR‐34a in the presence of PFT‐α (Figure 3) and inhibiting SIRT1 in the presence of miR‐34‐I (Figures. 4‐6). We previously studied the effect of SIRT1 activation by SRT2104 – a novel SIRT1 activator – on diabetic endothelial dysfunction.^5^ Activation of SIRT1 lowered endothelial ac‐P53 level that was increased under the HG/diabetic conditions.^5^ Therefore, P53, miR‐34a and SIRT1 may form a positive feedback loop in diabetic endothelial dysfunction (Figure 6D). Under HG/diabetic conditions, P53 and miR‐34a are predominant factors within this circuit, whereas SIRT1 is suppressed by miR‐34a. This inactivation of SIRT1 may lead to uncontrolled activation of P53 and overexpression of miR‐34a, accelerating the progression of diabetic endothelial dysfunction (Figure 6D). P53, miR‐34a and SIRT1 are key factors in diabetic endothelial dysfunction, as modulation of each of these factors produced a remarkable effect (Figures 2G,H; 3C,D; 4D,E; 5C‐F; 6B,C; 5). This suggests P53, miR‐34a and SIRT1 as important candidates for clinical intervention of diabetic endothelial dysfunction.

Previous studies have demonstrated that miR‐34a negatively regulates SIRT1 expression.^14^, ^15^, ^43^ Mechanistically, miR‐34a targets *Sirt1* mRNA without degrading *Sirt1* mRNA, inhibiting SIRT1 protein production.^15^, ^43^ In the present study, in order to investigate how miR‐34a regulates *Sirt1*expression, ECs were treated with the miR‐34a inhibitor (miR‐34a‐I) under HG condition. We observed that SIRT1 protein (Figure 4B), but not *Sirt1* mRNA (Figure 4A), was significantly decreased by miR‐34a‐I (Figure 4A,B, red columns). This result is in line with the previous findings,^43^ confirming that miR‐34a inhibits *Sirt1* gene expression at the translational level.

In the present study, HG induced a mild decrease in *Sirt1* mRNA in ECs (Figure 1E). However, HG resulted in a steep decrease in SIRT protein in ECs (Figure 1F). This differential regulation of the *Sirt1*gene expression under the HG condition indicates the involvement of translational regulation, where miR‐34a exerts its action (Figures 1D‐F; 3A,B; 5B,D; 13, 14). Nonetheless, the decrease in *Sirt1* mRNA level under the HG condition, although mild (Figure 1E), suggests that HG induces a transcriptional inhibition of the *Sirt1* gene.

We observed lower SIRT1 protein level in the HG + miR‐34a‐I + *Sirt1*‐siRNA group (Figure 4B) and lower SIRT1 activities in the HG + miR‐34a‐I + *Sirt1*‐siRNA and HG + miR‐34a‐I + EX527 groups (Figure 4C), as compared with the HG group. However, these did not significantly increase inflammation and oxidative stress (Figure 4D,E). One explanation could be that the HG condition already resulted in a very low basal level of SIRT1 protein (Figures 1F,2F). In other words, very few SIRT1 proteins were left under the HG condition. Hence, the decrease in SIRT1 protein level or activity, although statistically significant, might not be able to result in a remarkable exacerbation of inflammation and oxidative stress.

In the present study, VCAM‐1 and 4‐HNE were highly expressed not only in the intima, but also in the adventitia, under the diabetic condition (Figure 5E,F). The adventitia is known to play a role in vascular inflammation and oxidative stress.^44^, ^45^ The overexpression of VCAM‐1 in the adventitia could be caused by adventitial ECs which were generated during neovascularization.^46^ In addition, adventitial fibroblasts have been shown to produce substantial amounts of ROS in response to vascular injury.^44^ This observation should encourage future study of the role of adventitia in diabetic endothelial dysfunction and macrovascular complications.

MiR‐34a targets multiple mRNAs.^16^ MiR‐34a was reported to target *Notch* mRNA and exacerbate diabetic endothelial dysfunction^41^ in rats. In the present study, SIRT1 was found to mediate miR‐34a's pathogenic effect on diabetic endothelial dysfunction, because inhibition of SIRT1 abrogated miR‐34a‐I's protective effects both in vitro (Figure 4D,E) and in vivo (Figures 5E,F & 6B,C). SIRT1 positively regulated NOTCH in *Drosophila*,^47^ indicating a potential SIRT1/NOTCH pathway in protecting against diabetic endothelial dysfunction. However, more evidence has shown the negative regulatory effect of SIRT1 on NOTCH through deacetylation in ECs.^48^, ^49^ Moreover, numerous studies have demonstrated the pathogenic role of NOTCH in diabetic endothelial dysfunction.^50^, ^51^ Therefore, it is needed to investigate whether or not NOTCH mediates miR‐34a's action on diabetic endothelial dysfunction by gene silencing/overexpression in future studies.

Collectively, the present study has found an unhindered P53/miR‐34a/SIRT1 pathway that plays a critical role in diabetic endothelial dysfunction, providing novel strategies for future management of diabetic macrovascular diseases.

## CONFLICT OF INTEREST

The authors confirm that there are no conflict of interests.

## AUTHOR CONTRIBUTION

Hao Wu conceived and designed the project. Junduo Wu, Wenzhao Liang, Yueli Tian, Fuzhe Ma, Wenlin Huang, Ye Jia, Ziping Jiang and Hao Wu researched and interpreted the data. Hao Wu, Ziping Jiang and Junduo Wu wrote the manuscript. Hao Wu, Ziping Jiang, Junduo Wu, Wenzhao Liang, Yueli Tian, Fuzhe Ma, Wenlin Huang and Ye Jia discussed, reviewed and revised the manuscript. Hao Wu, Ziping Jiang, Junduo Wu and Wenzhao Liang provided funding. All the authors approve the version to be published.
